# Factors Affecting Intensive Care Units Nursing Workload

**DOI:** 10.5812/ircmj.20072

**Published:** 2014-08-05

**Authors:** Mohammadkarim Bahadori, Ramin Ravangard, Mehdi Raadabadi, Seyed Masod Mosavi, Mohammad Gholami Fesharaki, Fardin Mehrabian

**Affiliations:** 1 Health Management Research Center, Baqiyatallah University of Medical Sciences, Tehran, IR Iran; 2 School of Management and Medical Information Sciences, Shiraz University of Medical Sciences, Shiraz, IR Iran; 3 Health Services Management Research Center, Kerman University of Medical Sciences, Kerman, IR Iran; 4 Students Scientific Research Center (SSRC), Tehran University of Medical Sciences, Tehran, IR Iran; 5 Hospital Management Research Center, Iran University of Medical Sciences, Tehran, IR Iran; 6 Biostatistic Departmant, Faculty of Medical Sciences, Tarbiat Modares University, Tehran, IR Iran; 7 School of Health, Guilan University of Medical Sciences, Rasht, IR Iran

**Keywords:** Confirmatory Factor Analysis, Nurses, Workload, Intensive Care Units, Iran

## Abstract

**Background:**

The nursing workload has a close and strong association with the quality of services provided for the patients. Therefore, paying careful attention to the factors affecting nursing workload, especially those working in the intensive care units (ICUs), is very important.

**Objectives:**

This study aimed to determine the factors affecting nursing workload in the ICUs of the hospitals affiliated to Tehran University of Medical Sciences.

**Materials and Methods:**

This was a cross-sectional and analytical-descriptive study that has done in Iran. All nurses (n = 400) who was working in the ICUs of the hospitals affiliated to Tehran University of Medical Sciences in 2014 were selected and studied using census method. The required data were collected using a researcher–made questionnaire which its validity and reliability were confirmed through getting the opinions of experts and using composite reliability and internal consistency (*α* = 0.89). The collected data were analyzed through exploratory factor analysis (EFA), confirmatory factor analysis (CFA) and using SPSS 18.0 and AMOS 18.0.

**Results:**

Twenty-five factors were divided into three major categories through EFA, including structure, process, and activity. The following factors among the structure, process and activity components had the greatest importance: lack of clear responsibilities and authorities and performing unnecessary tasks (by a coefficient of 0.709), mismatch between the capacity of wards and the number of patients (by a coefficient of 0.639), and helping the students and newly employed staff (by a coefficient of 0.589).

**Conclusions:**

The nursing workload is influenced by many factors. The clear responsibilities and authorities of nurses, patients' admission according to the capacity of wards, use of the new technologies and equipment, and providing basic training for new nurses can decrease the workload of nurses.

## 1. Background

Nowadays, the employees' physical and mental health is as important as production and productivity for any organization (1). The physically and mentally healthy employees can increase organizational productivity and, therefore, provide more effective services. They play a key role in the continued success of the organization and in achieving its short-term objectives (2). The researchers of the fields of management and organizational psychology have concluded that job stress has an important influence on the reduction of organizational effectiveness (3, 4).

According to the results of the studies on the job stress, nurses have the greatest job stress (5-7). Although the low level of job stress in the modern nursing leaves no detrimental effects, in the long-term it can have harmful effects, including cardiovascular diseases, respiratory diseases, etc. and, ultimately, can reduce the nurses' quality of life. Work environments such as hospitals and their operating rooms have considerable effects on the employees' mental health because of their stressful nature. Therefore, it is suggested that the work environments of such employees should be changed every few years (8, 9).

The work environment of nurses has changed significantly in the past few years which is the result of some factors such as health reforms, hospital renovation, the shortage of nurses in the face of rapid technological advances and patients' expectations to receive high-quality services (10, 11). On the other hand, the increasing number of patients and lack of nursing personnel are two main reasons for the nurses and patients' dissatisfaction with the provided services (12). Currently, the nursing profession has changed into a complex process, and the existence of a person with high decision-making power to diagnose the severity of diseases is necessary (13, 14). The results of a study conducted on a group of nurses in 2007 showed that 50% of them assessed their job as a tough job and 12% of them assessed it as a hard one a major part of which was not related directly to the medical services. However, the recent reports indicate a decrease in the nurses' health benefits during patients' treatment, because of their high workload (15). Therefore, the nurses' work pressure and workload are not only determined by a functional framework; but also many cognitive factors affect them which shows the complexity of their tasks (16).

de Cordova et al. in their study stated that a nurse's workload was not only determined through a specific package of guidance; but many factors, including cognitive factors and the complexity of nurses' work environment, had an important role in accurate estimating of nurses' workload (17). In fact, the work pressure is considered as a function of time influenced by factors such as the level of complexity, and the number of services provided (18). Although many studies have been conducted on workload, a clear definition of the term "workload" has not been provided by the researchers yet (19). However, the workload can be considered as a biopsychosocial factor, so that any increase in the workload not only increases absenteeism, but also is a factor results in the employees' withdrawal from their work environment and, therefore, changes in their career and professional lives (10).

This factor is more important in the intensive care units (ICUs) compared to other wards and units because a large number of patients are hospitalized in the ICUs in each year. These patients need to receive special care such as ventilation, injections, prescribing antibiotics, etc. for a long time which highlights the role of nurses, especially after the physicians' orders were prescribed (20). In the studies conducted in the ICUs of hospitals, two factors have been identified as the main barriers to measure the workload: the nurses' interactions with the patients and the existence of many qualitative indicators in the process of providing care for patients. Moreover, two factors have been considered as the important factors affecting the failure to allocate sufficient time to each patient by nurses: the increase in the load of services provided to patients, and the shortages of nursing personnel (10, 21).

In recent years, the numbers of ICUs beds have increased (22, 23). Many researchers believe that working in this unit is an important source of social and psychological pressure and stress for employees. The stressful factors are poor lighting, excessive noise, a large number of specialists and medical equipment, high patient mortality, the lack of tangible outcomes of services provided by nurses, and the need for proper decision-making (21). Therefore, recognizing, categorizing and prioritizing these threats in order to formulate and implement appropriate policies and interventions, as well as developing the suitable educational topics and training syllabus require sufficient knowledge of occupational health, which has been neglected over the years despite the development of this profession (24-27). The understanding and knowledge of factors affecting nursing workload can change the nurses' attitudes and perceptions towards this area (19).

## 2. Objectives

The present study aimed to determine the factors affecting the workload of nurses working in the ICUs of hospitals affiliated to Tehran University of Medical Sciences.

## 3. Materials and Methods

This was a cross-sectional and analytical-descriptive study.

### 3.1. Setting

The study was conducted between January 2014 and February 2014 in hospitals affiliated to Tehran University of Medical Sciences in Tehran, Iran. All studied hospitals were state-run and teaching hospitals affiliated to Tehran University of Medical Sciences among which four hospitals were general and eight hospitals were specialty hospitals, including women, children, ENT, dermatology, and eye hospitals. Imam Khomeini hospital had the highest number of beds (1230), staff (2023) and wards (41). On the other hand, Roozbeh Hospital had the lowest number of beds (69), staff (130) and wards (4).

### 3.2. Samples

All nurses (n = 400) were working in three shifts (150 nurses in the morning shift, 150 nurses in the afternoon shift, and 100 nurses in the night shift) of the intensive care units (ICUs) of the teaching hospitals affiliated to Tehran University of Medical Sciences in 2014. They were selected and studied using census method. The inclusion criteria were those nurses working in the ICUs for at least 6 months, and the only exclusion criteria was those nurses who didn't want to participate in the study.

### 3.3. Data Collection

The required data were collected through the review of the literature obtained from all related databases, previously conducted studies, as well as the field data collection using a researcher-made questionnaire to measure the importance of each factor affecting nurses' workload.

This questionnaire consisted of two sections. The first section included 7 items about the studied nurses' demographic characteristics, including age, sex, marital status, education level, job experience, employment status, and position, and the second section included 25 items about the factors affecting the workload of nurses. A five-point Likert scale was used for each factor whereby 1 refers to strongly disagree and 5 as strongly agree. For data gathering, one of the researchers referred to the hospitals in the morning, afternoon and night shifts. The head nurse of each ward helped the researcher for data gathering. One week after distributing the questionnaires among the nurses, the researcher collected the completed ones. The response rate was 100%.

### 3.4. Validity and Reliability

The validity of the questionnaire was confirmed through getting the opinions of faculty members of nursing school and calculating content validity index (CVI = 0.75) and content validity ratio (CVR = 0.74) of the questionnaire. Also its reliability was confirmed using composite reliability and internal consistency (α = 0.89). The percent of participants with scores at the ceiling (score of 5) and floor (score of 1) were calculated for each of the scales. The ceiling and floor effects were less than 20% to ensure that the scale takes the full range of potential responses within the target population and that the changes can be detected over time.

### 3.5. Data Analysis

The collected data were analyzed using exploratory factor analysis (EFA) to determine the main components affecting workload of nurses and confirmatory factor analysis (CFA) for confirming the model. In addition, the principal components analysis with varimax rotation was used to identify the load of each factor on the main components. All analyses were carried out using SPSS 18.0 and AMOS 18.0.

### 3.6. Ethical Consideration

This study was conducted only on nurses. An approval for conducting this study was obtained from the ethics committee of Baqiyatallah University of Medical Sciences (ethical code: CH/7018/99). The verbal consents were obtained from all nurses participating in this study, and all of them were assured of the confidentiality of their responses.

## 4. Results

The results showed that the mean age of the participants was 32.9 y (SD = 7.14), and most of them were female (91%), married (63.8%), employed officially (55.3%), nurses (84.5%), had a bachelor's degree (91.3%), and had less than 6 years job experience (41%) (Table 1).

Furthermore, the results showed that KMO > 0.7 and P value (Bartlett's test) < 0.001. Therefore, the collected data were suitable for performing exploratory factor analysis (Table 2).

As mentioned earlier, the principal components analysis with varimax rotation was used to identify the most important components affecting the nurses' workload and the level of loading each factor had on the main components. As it can be seen in the table of total explained variance (Table 3), these questions formed a total of 3 factors which explained 41.88% of the variance of the components.

In order to obtain a meaningful structure of the factor loadings, they were extracted based on the common methods and direct oblimin rotation which were transferred to the new axes with a non-perpendicular angle relative to each other. Therefore, in exploratory factor analysis, 25 factors were divided into three major components: structure, process, and activity. The following factors among the structure, process and activity components had the greatest importance: lack of clear responsibilities and authorities and performing unnecessary tasks (by a coefficient of 0.709), mismatch between the capacity of wards and the number of patients (by a coefficient of 0.639), and helping the students and newly employed staff (by a coefficient of 0.589) (Table 4).

These components were as follows in order of importance:

1. Structure with 18 factors (variance = 22.065% and Eigenvalue = 5.373)
2. Process with 5 factors (variance = 14.66% and Eigenvalue = 3.813)
3. Activity with 2 factors (variance = 5.152% and Eigenvalue = 1.340)

The components were calculated as follows:

Component of structure = Mean (Q 1, Q 3, Q 7, Q 8, Q 9, Q 11, Q 13, Q 15, Q 16, Q 17, Q 18, Q 19, Q 20, Q 21, Q 22, Q 23, Q 24, and Q 25)

Component of process = Mean (Q 2, Q 5, Q 6, Q 12, and Q 13)

Component of activity = Mean (Q 4 and Q 10)

Confirmatory factor analysis, also, was used to specify the studied samples data and responses in conformity to the suggested structure. The results of the fitness indices for the model have been shown in the Table 5 indicating the acceptability of studied index.

**Table 1.** Demographic Characteristics of The Studied Nurses

Variables	No. (%)
**Sex**	
Male	36 (9)
Female	364 (91)
**Marital Status**	
Single	145 (36.2)
Married	255 (63.8)
**Education Level**	
Diploma	5 (1.3)
Associate Degree	5 (1.3)
Bachelor's Degree	365 (91.3)
Master's Degree	25 (6.3)
**Job Experience , y**	
< 6	164 (41)
6-10	145 (36.3)
11-15	45 (11.3)
16-20	46 (11.5)
**Employment Status**	
Treaty Employees	119 (29.8)
Official and Formal Employees	221 (55.3)
Contract Employees	60 (15)
**Position**	
Matron and Nurse Managers	16 (4)
Head Nurse	38 (9.5)
Supervisor	8 (2)
Nurse	338 (84.5)

**Table 2.** KMO and Bartlett's Test

**Kaiser-Meyer-Olkin Measure of Sampling Adequacy**	0.902
**Bartlett's Test of Sphericity**	
Approx. Chi-Square	3296.851
df	325
P value	< 0.001

**Table 3.** Total Explained Variance of The Studied Components ^a^

Components	Initial Eigen Values			Extraction Sums of Squared Loadings		
	Eigen Value	Percent of Variance	Cumulative (%)	Eigen Value	Percent of Variance	Cumulative (%)
**Factor 1**	7.602	29.239	29.239	7.602	29.239	29.239
**Factor 2**	1.948	7.492	36.732	1.948	7.492	36.732
**Factor 3**	1.339	5.152	41.883	1.339	5.152	41.883

^a^ Extraction Method: Principal Component Analysis

**Table 4.** Rotated Factor Matrix ^a^

Question	Factors	Component 1 Structures	Component 2 Process	Component 3 Activity
**Q 1**	Lack of clear responsibilities and authorities and performing unnecessary tasks	0.709		
**Q 2**	Mismatch between the capacity of wards and the number of patients		0.693	
**Q 3**	Large number and variety of tasks assigned to the nurses	0.675		
**Q 4**	Helping the students and newly employed staff			0.589
**Q 5**	Lack of proper system to help those units which are suffering from a shortage of personnel		0.561	
**Q 6**	Unanticipated and unscheduled admissions and discharge		0.509	
**Q 7**	Troublesome clinical principles and rules in the wards	0.615		
**Q 8**	Improper design of the hospital and its wards	0.552		
**Q 9**	Shortages of secretaries, logistics staff and supervisors	0.494		
**Q 10**	Weight gain in some patients and difficulty in moving them			0.454
**Q 11**	Lack of social and technical support for nurses	0.415		
**Q 12**	Lack of teamwork in nursing processes		0.326	
**Q 13**	Lack of trained teams and staff to transport patients in the hospital	0.463		
**Q 14**	Spending too much time in the meetings of different nursing wards		0.537	
**Q 15**	Heterogeneous skills of staff in a nursing team	0.478		
**Q 16**	Time needed to control the tools and materials	0.539		
**Q 17**	Poor personal work	0.596		
**Q 18**	Lack of integration of nursing tools and materials	0.596		
**Q 19**	Limited number of single rooms in the wards	0.523		
**Q 20**	Excessive compulsory education	0.634		
**Q 21**	Excessive CPRs in the wards compared to their facilities and capacities	0.674		
**Q 22**	Lack of hospital equipment in emergencies	0.655		
**Q 23**	Excessive research and quality improvement activities in the hospital	0.605		
**Q 24**	Lack of stability and consistency in the hospital organization	0.670		
**Q 25**	Lack of a trained team for transfering patients to other hospitals	0.681		
**Mean ± SD**		3.55 ± 0.61	3.56 ± 0.65	3.23 ± 0.84

^a^ Extraction Method: Principal Component Analysis

**Table 5.** Indicators of Confirmatory Factor Analysis model

Goodness of Fit index	Acceptable Values	Observed Values
**X2**	-	550.5
**df**	-	215
**X2/df**	1 < X < 3	2.56
***P*** ** value**	< 0.05	0.000
**RMSEA**	< 0.08	0.063
**PGFI**	> 0.6	0.695
**PCFI**	> 0.6	0.742

## 5. Discussion

The present study aimed to identify the most important factors affecting the nursing workload and the level of each factor loading on the main components. Studies performed in the last decade have indicated that, because of the complexity of workload concept and the difficulty of calculating work staff needed for a ward (28), conducting reliable studies on the workload of nurses can help matrons and nurse managers to make evidence-based decisions (17). Gaba and Lee also believed that assessing the workload of the health care providers is necessary because many stressors may affect the management of the high volume of their work. In addition, the high volume of their regulatory tasks and obligations make it difficult to identify and respond to the emergencies (29).

The present study was conducted on the nurses working in the ICUs of the teaching hospitals. The ICU nurses are usually faced with the most severe emotional issues and problems and should make important decisions with regard to the patients' lives, as well as continuously meet the demands of patients and their relatives. Therefore, they are confronted with the heavy workload which is one of the most important factors influencing their stresses (30-32). This fact doubles the necessity of paying attention to the workload of ICU nurses.

In the present study, 25 factors affecting the nursing workload were studied from the viewpoint of nurses working in the ICU by a questionnaire. Myny et al. in a review of the literature investigated 20 non-direct factors affecting nursing workload and classified them into five categories based on their level of effect: the hospital and ward, nursing team, individual nurse, patient and family, and meta-characteristics (33). Myny et al. in another study (34) investigated 28 factors affecting nursing workload from the perspective of nurses which were very similar to the factors studied in the present study. Furthermore, Gurses and Carayon in their study on the nurses' workload showed that workplace conditions, tools and equipment, the relationship between employees and information exchange, transporting the patient in the hospital, patients-related factors, helping colleagues, getting help from colleagues, and working in the teaching hospitals had effects on ICUs nurses' workload (31).

Because the structure and content of nurses' workload have a wide range and extend beyond a predetermined framework; there are many factors that can affect the workload of nurses and different methods to measure it. In the present study, factors which had greater importance according to the results of other studies and nursing managers' viewpoints were selected and studied. 

Among 18 factors of the structure component, the lack of clear responsibilities and authorities and performing unnecessary tasks, lack of a trained team for transferring patients to other hospitals, and lots of various tasks assigned to the nurses had the greatest effects on the nurses' workload. Tucker and Spear (28), Redding and Robinson (35), and Cornell et al. (36) in their studies found that nurses' work interruptions, lack of clear responsibilities and authorities, and performing unnecessary tasks were the most important factors affecting nurses' workload. Myny et al. also in their study concluded that, among the studied factors, the lack of clear responsibilities and authorities and performing unnecessary tasks, the mismatch between the capacity of wards and the number of patients, and the large number and variety of tasks assigned to the nurses were the important factors influencing the nurses' workload (34).

Among the factors of the process component, the mismatch between the capacity of wards and the number of patients, and among the factors of activity component, helping the students and newly employed staff had the greatest effects on the nurses' workload. Beswick et al. (37) and Duffield et al. (38) in their studies indicated that the accurate measurement of nurses' work hours and doing work in terms of integrated admissions and discharge in order to establish a match between the capacity of the wards, and the number of patients were necessary. The results of Aiken et al.’s study showed that only less than 42% of nurses in America, Canada, England, and Scotland believed that there were enough nurses to provide high-quality services and also there were enough employees to do the work (39).

In all studied hospitals, the mismatch between the capacity of wards and the number of patients admitted to these patients has been expressed by nurses as one of the factors affecting their workload, which causes the problems in the nurses' performances, increases stress and medical errors, reduces the quality of care, and ultimately decreases patients' satisfaction. Therefore, developing strategies to reduce environmental disturbances, utilizing new technologies and equipment, and redefining the role of nurses should be considered by researchers (40) so that the workload of nurses to be reduced. Moreover, the results of recent studies have indicated that the high ratio of patients to nurses, the mismatch between the capacity of wards and the number of patients, and subsequently, the increase in the nurses' workload are some of the main reasons for nurses' turnover (41, 42). Therefore, if the mentioned factors such as environmental and physical conditions of wards, administrative and clinical processes, and other factors increasing the workload of the nurses are being overlooked, they will cause problems such as suboptimal patient care, difficulties in making correct decisions by nurses and medical team, weakened nurse-patient relationships, distorted nurse-physician relationships, and ultimately increased medical staff's burnout and job dissatisfaction.

This study (investigating factors affecting nurses' workload) had been conducted on the nurses working in the ICUs in Iran for the first time in which a useful method had been developed for measuring nurses' workload. The limitation of this study was that this instrument could only be used for measuring the workload of nurses working in the ICUs not in other wards. 

Overall, the results of the present study and other studies aforementioned show that the nurses' workload is influenced by many factors on which further studies are needed. These factors, which have close and strong associations with the nurses' working conditions and duties, can prevent or facilitate their performances. On the other hand, the high workload consequences such as nurses' non-compliance with care guidelines, nurses' inadequate monitoring of patients, etc. can be at the patient level (including lower quality of care and safety), the patients' relatives level (including dissatisfaction with the care provided), and the nurse’s level (including lower nurse’s quality of working life). However, the clear responsibilities and authorities of nurses, patients' admission based on the capacity of wards, the use of the modern technologies and equipment, providing basic training for new nurses, etc. can decrease the nurses' workload.
